# Neuroendocrine and cellular mechanisms in stress resilience: From hormonal influence in the CNS to mitochondrial dysfunction and oxidative stress

**DOI:** 10.1111/jcmm.18220

**Published:** 2024-03-20

**Authors:** Arghya Bhattacharya, Manas Chakraborty, Ananya Chanda, Taha Alqahtani, Ajoy Kumer, Bikram Dhara, Moitreyee Chattopadhyay

**Affiliations:** ^1^ Department of Pharmacology Calcutta Institute of Pharmaceutical Technology and AHS Uluberia West Bengal India; ^2^ Department of Pharmaceutical Biotechnology Calcutta institute of pharmaceutical technology and AHS Uluberia West Bengal India; ^3^ Department of Pharmaceutical Science Adamas University Barasat West Bengal India; ^4^ Department of Pharmacology, College of Pharmacy King Khalid University Abha Saudi Arabia; ^5^ Department of Chemistry College of Arts and Sciences, IUBAT‐International University of Business Agriculture and Technology Dhaka Bangladesh; ^6^ Center for Global Health Research Saveetha Medical College and Hospital, Saveetha Institute of Medical and Technical Sciences Chennai India; ^7^ Department of Health Sciences Novel Global Community and Educational Foundation Hebersham New South Wales Australia; ^8^ Department of Pharmaceutical Technology Maulana Abul Kalam Azad University of Technology Kolkata West Bengal India

**Keywords:** HPA axis, oxidative stress, resilience, stress, synthetic hormone

## Abstract

Recent advancements in neuroendocrinology challenge the long‐held belief that hormonal effects are confined to perivascular tissues and do not extend to the central nervous system (CNS). This paradigm shift, propelled by groundbreaking research, reveals that synthetic hormones, notably in anti‐inflammatory medications, significantly influence steroid psychosis, behavioural, and cognitive impairments, as well as neuropeptide functions. A seminal development in this field occurred in 1968 with McEven's proposal that rodent brains are responsive to glucocorticoids, fundamentally altering the understanding of how anxiety impacts CNS functionality and leading to the identification of glucocorticosteroids and mineralocorticoids as distinct corticotropic receptors. This paper focuses on the intricate roles of the neuroendocrine, immunological, and CNS in fostering stress resilience, underscored by recent animal model studies. These studies highlight active, compensatory, and passive strategies for resilience, supporting the concept that anxiety and depression are systemic disorders involving dysregulation across both peripheral and central systems. Resilience is conceptualized as a multifaceted process that enhances psychological adaptability to stress through adaptive mechanisms within the immunological system, brain, hypothalamo–pituitary–adrenal axis, and ANS Axis. Furthermore, the paper explores oxidative stress, particularly its origin from the production of reactive oxygen species (ROS) in mitochondria. The mitochondria's role extends beyond ATP production, encompassing lipid, heme, purine, and steroidogenesis synthesis. ROS‐induced damage to biomolecules can lead to significant mitochondrial dysfunction and cell apoptosis, emphasizing the critical nature of mitochondrial health in overall cellular function and stress resilience. This comprehensive synthesis of neuroendocrinological and cellular biological research offers new insights into the systemic complexity of stress‐related disorders and the imperative for multidisciplinary approaches in their study and treatment.

## 1 INTRODUCTION

For decades, the scientific consensus held that hormones primarily interacted with receptors in peripheral tissues and had a limited effect on the CNS.^1^ This view has evolved significantly with recent research indicating that synthetic hormones, such as those found in anti‐inflammatory medications, can profoundly influence both steroid psychosis and cognitive and behavioural impairments.^2^ This development highlights the complex interplay between hormonal activities and CNS functions. Initially, in the early 1960s, neuropeptides like vasopressin, cholecystokinin^3^ were thought to have minimal impact on the peripheral endocrine system. However, subsequent studies have demonstrated the significant role of hormones in influencing behaviour and cognition, suggesting their physiological effects on various CNS regions.^4^ A critical turning point came in 1968 with the hypothesis that rodent brains respond to glucocorticoids, a key element in the stress response.^5^ This led to the recognition that anxiety can significantly alter CNS functionality. Further research differentiated glucocorticosteroids and mineralocorticoids as distinct corticotropic receptors.^6^ Studies found that cortisol and corticosterone have an affinity for glucocorticosteroid receptors much lower than for mineralocorticoid receptors.^6^ Notably, certain brain regions, such as the hippocampus, contain both types of receptors, indicating a nuanced interaction of hormonal pathways within the CNS.^6^ Research on human stress resilience has expanded since its initial focus on at‐risk children in the 1970s.^7^ Resilience is defined as the ability to maintain normal physiological, developmental and behavioural states under extreme stress. Numerous studies document instances of individuals, both adults and children, showing remarkable emotional stability and lack of behavioural disorders under significant psychological stress.^8^, ^9^, ^10^, ^11^, ^12^ This resilience suggests an inherent capacity in most individuals to avoid conditions like depression and anxiety under stress, challenging the notion that extraordinary abilities or coping mechanisms are essential for stress adaptation.^7^, ^12^

The concept of ‘allostasis’ has been pivotal in understanding the biological mechanisms behind resilience.^13^ Allostasis involves dynamic systemic responses to stressors to maintain homeostasis. However, when allostasis becomes prolonged or fails to end with the stressor, once protective systems can become pathological, leading to ‘allostatic load’ and increasing the risk of physical and mental health issues, including depression and anxiety.^13^, ^14^ The impact of stress‐related disorders, such as depression and anxiety, is significant. Major depressive disorder (MDD), for instance, affects about one in six individuals in the United States, with only a fraction achieving full remission with standard antidepressant treatments.^15^, ^16^ Untreated, MDD can evolve into a chronic, increasingly debilitating condition.^17^ Understanding the aetiology of depression remains complex. Insights into the adaptive, allostatic processes that protect most individuals from psychopathology could lead to new therapeutic strategies for those at greater risk. The growing recognition of anxiety and depression as ‘whole body’ disorders involves dysregulation of various peripheral and central systems. Similarly, resilience likely arises from effective allostatic processes in the immune system, brain and the hypothalamo–pituitary–adrenal (HPA) axis.^13^

**FIGURE 1:**
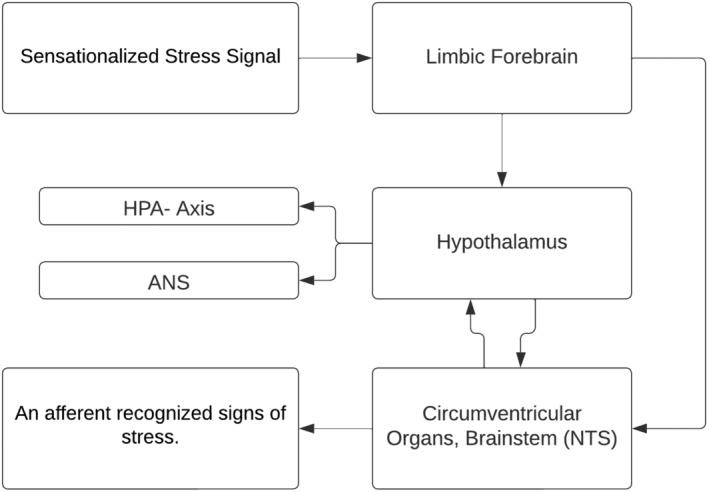
The interaction between the hypothalamic–pituitary–adrenal (HPA) axis, the autonomic nervous system (ANS) and the central nervous system (CNS) in enhancing adaptive physiological responses to stress.

In this review we provide a comprehensive overview of the intricate neuroendocrine and cellular mechanism involved in stress resilience. It explores the interaction between the hypothalamic–pituitary–adrenal (HPA) axis, the autonomic nervous system (ANS) and the central nervous system (CNS) in increasing adaptive physiological responses to stress. The article highlights recent animal model studies focused on developmental stress scenarios and maternal care effects on offspring's neuroendocrine and epigenetic changes, elucidating the molecular factors contributing to enhanced glucocorticoid receptor expression and consequently greater stress resilience. It also examines the mesocorticolimbic reward circuitry dynamics in the pathophysiology of mood disorders like depression and anxiety. Additionally, the article discusses oxidative stress arising from mitochondrial reactive oxygen species production and the potential of targeted PROTAC therapies to selectively degrade proteins involved in abnormal ROS generation or antioxidant response. Furthermore, it explores the benefits of traditional herbal medicine and specific nutrients in mitigating anxiety and depression symptoms through their effects on various neurotransmitters and neuropeptides. Overall, the article emphasizes an integrated, systemic approach considering the neuroendocrine system, CNS pathways, cellular biology and psychological factors to advance our understanding of stress resilience and develop more effective therapeutic strategies (Figure. 1).

## 2 NEUROENDOCRINE PATHWAYS IN STRESS RESILIENCE: AN IN‐DEPTH ANALYSIS

**FIGURE 2:**
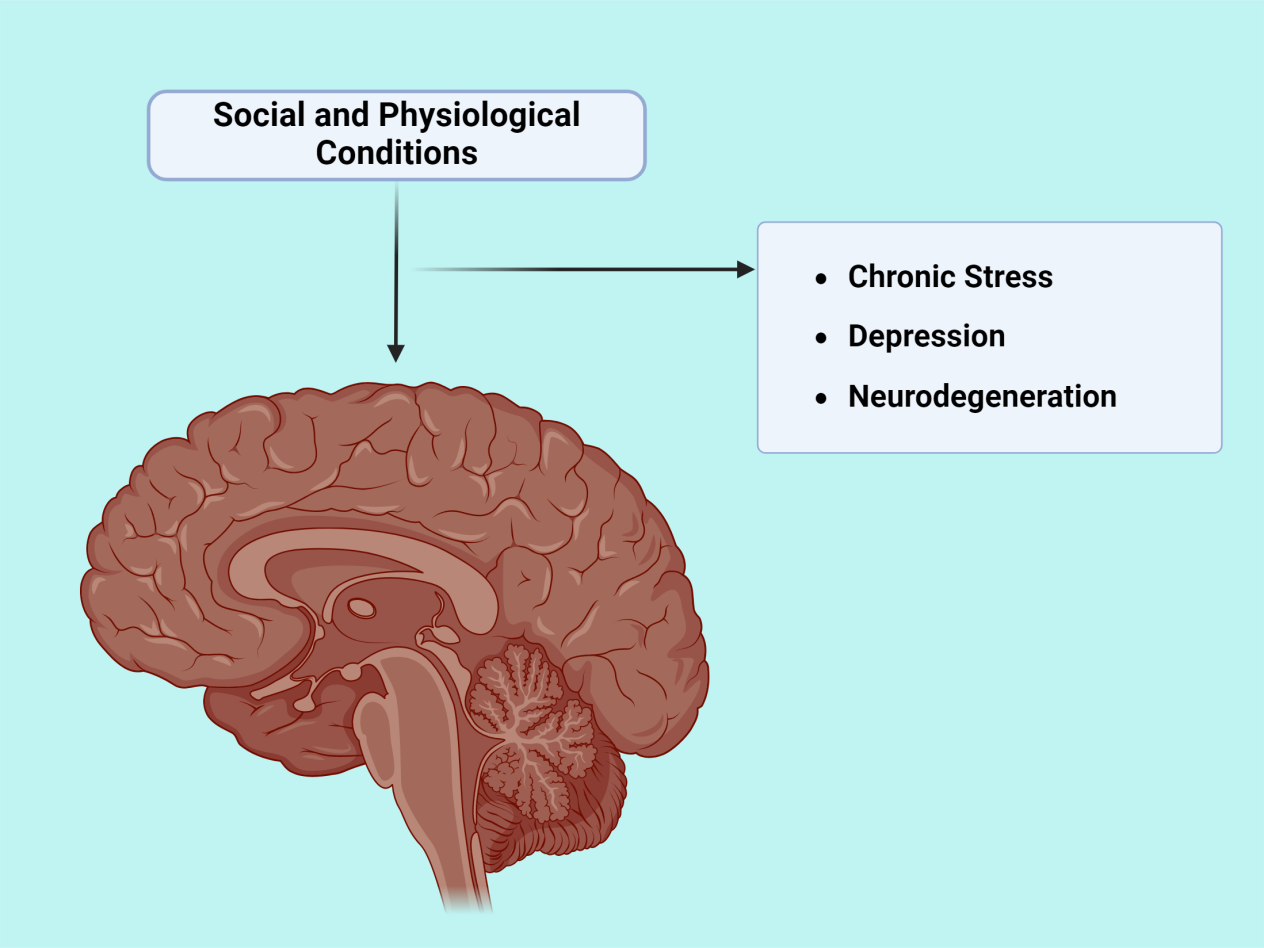
Features a human brain with highlighted neural pathways, surrounded by symbols representing stress factors and resilience elements.

The neuroendocrine mechanisms underlying resilience to stress involve a complex network of neuronal pathways, prominently including the hypothalamo–pituitary–adrenal (HPA) axis, and extending to various regions of the brainstem, forebrain and hypothalamus, which collectively orchestrate the autonomic nervous system (ANS) response.^16^, ^17^, ^18^ This intricate system plays a critical role in mounting an appropriate physiological response to stress. During a stress response, the spinal cord activates preganglionic sympathetic neurons. These neurons extend to pre‐ and para‐vertebral ganglionic neurons, which in turn stimulate the sympathetic nerve fibres. Depending on their termination points, these fibres can target visceral organs, cardiovascular systems or the adrenal medulla. The activation of these fibres results in the release of noradrenaline and adrenaline, key neurotransmitters in stress response, from the adrenal medulla. The effects of this activation are multifaceted, including alterations in heart rate and vasoconstriction, both of which are hallmarks of the ANS's engagement during stress. Concurrently, stress triggers the HPA axis, initiating a cascade of hormonal releases (Figure 2).

**FIGURE 3:**
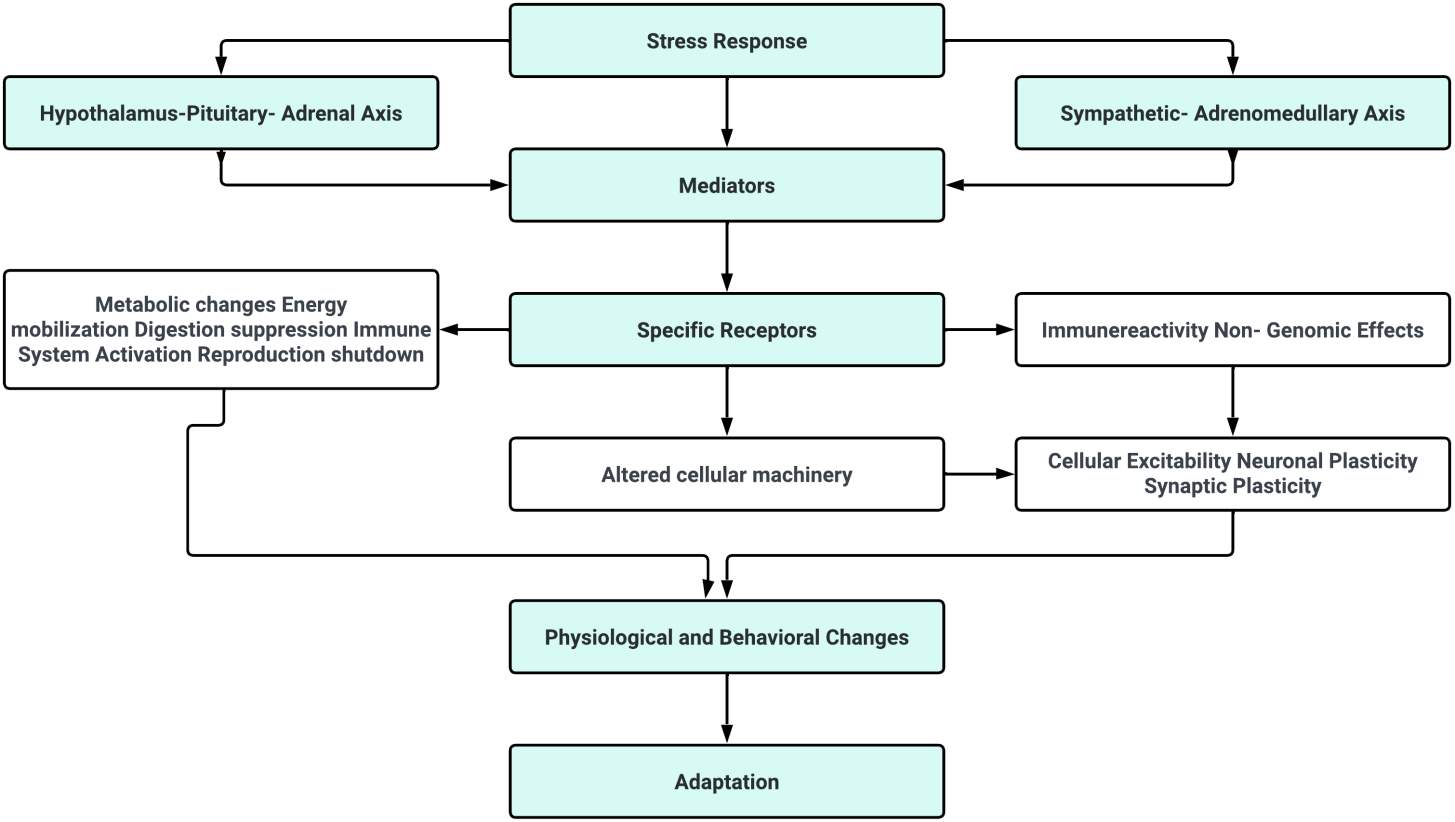
Processing and coping of stress system.^18^, ^19^

The process begins in the paraventricular nucleus (PVN) of the hypothalamus, where neurons release corticotropin‐releasing factor (CRF) and arginine vasopressin (AVP) into the portal circulation via the median eminence. This release stimulates the anterior pituitary gland, leading to the production of adrenocorticotropic hormone (ACTH). ACTH, in turn, prompts the adrenal cortex to produce and release glucocorticoids, primarily cortisol in humans. These glucocorticoids play a pivotal role in mobilizing energy reserves during stress, influencing various metabolic processes. The interplay between the ANS and the HPA axis is a crucial aspect of the stress response, illustrating a complex and dynamic communication network. This interaction ensures a coordinated and efficient response to stress, balancing the immediate sympathetic activation with the more prolonged HPA axis activation. Understanding this interplay is key to comprehending how the body maintains resilience in the face of acute and chronic stressors (Figure 3).

## 3 INFLUENCE OF NEUROENDOCRINE SYSTEMS ON STRESS RESILIENCE AND MOOD DISORDERS

**FIGURE 4:**
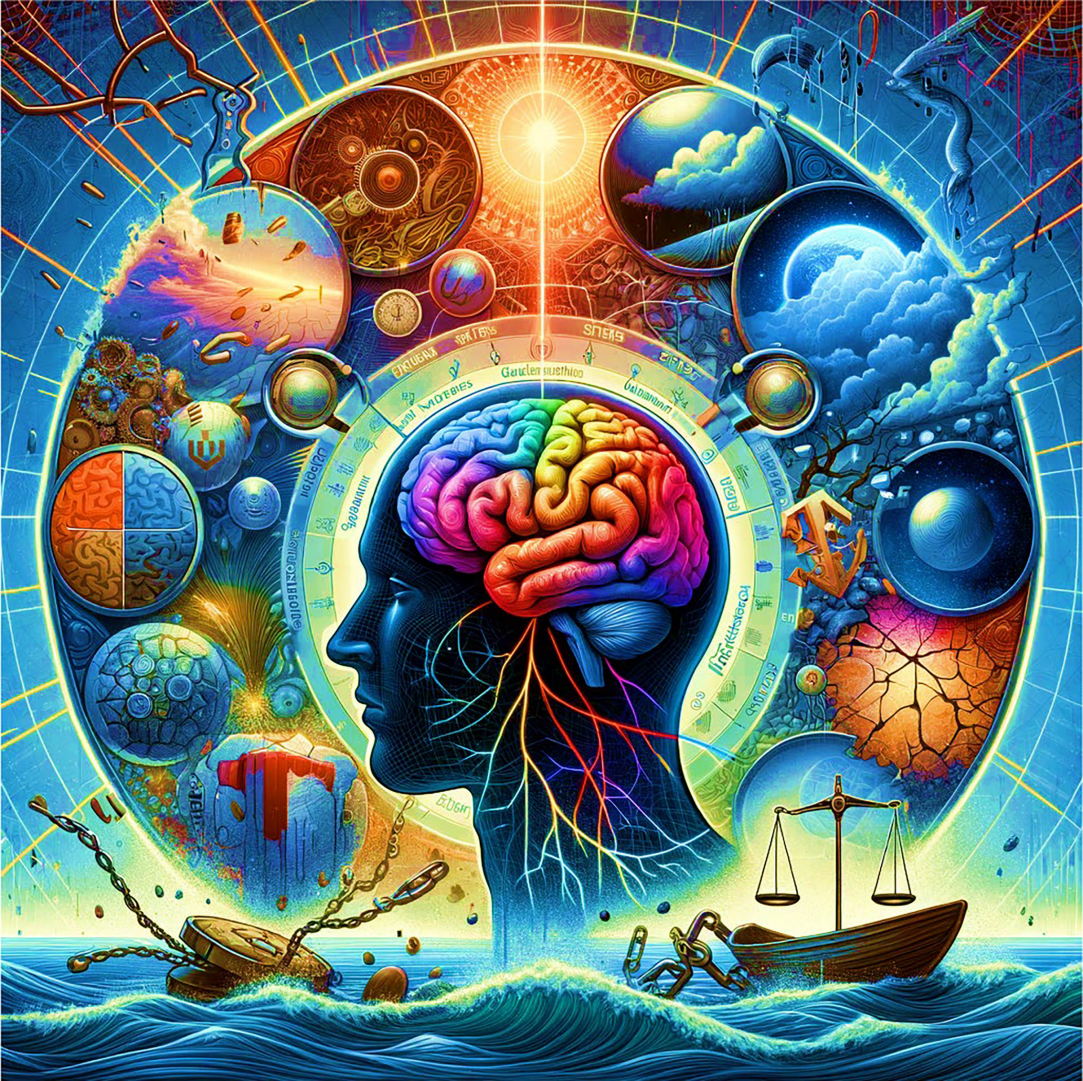
Features a human brain with various neuroendocrine systems highlighted, surrounded by symbolic representations of stress resilience and mood disorders (Created with DALL.E).

The interplay between the neuroendocrine systems, particularly the sympathetic nervous system and the adrenal cortex, plays a pivotal role in stress resilience and the pathophysiology of mood disorders (Figure 4). The sympathetic nervous system directly innervates the adrenal cortex, which in turn controls the release of glucocorticoids. These glucocorticoids, such as cortisol, significantly influence the autonomic nervous system (ANS)‐dependent stress responses, including vasoconstriction.^20^ While much of the evidence linking these systems to human resilience against mood disorders like major depressive disorder (MDD) and post‐traumatic stress disorder (PTSD) remains correlative, there are notable observations of modulation in these conditions.^20^ Emerging therapeutic approaches for PTSD include high‐dose glucocorticoid administration following exposure to traumatic stress. This intervention is hypothesized to mitigate hyperactive fear responses and inhibit the consolidation of fear memories.^20^ Clinical evidence supporting this approach has been observed in critically ill patients and combat veterans.^21^, ^22^ Furthermore, the role of neuroendocrine biomarkers, such as neuropeptide Y (NPY) and dehydroepiandrosterone (DHEA), in stress resilience is gaining attention. DHEA, released alongside cortisol by the adrenal cortex in response to stress, may counterbalance the effects of glucocorticoids.^23^ A negative correlation between DHEA levels and the severity of PTSD symptoms in combat veterans suggests DHEA's potential protective role in high‐stress situations.^23^ NPY, released concurrently with noradrenaline from sympathetic nerves, shows a positive correlation with enhanced performance under interrogation and a negative correlation with dissociative symptoms in soldiers undergoing combat training.^24^ The interaction between the hypothalamo–pituitary–adrenal (HPA) axis and the hypothalamic pituitary gonadal axis, sharing several neuronal circuits and structures, underlines the significant influence of reproductive hormones on stress sensitivity and resilience. Gender differences in the prevalence of mood disorders dysthymia, bipolar disorder^25^ emerging from puberty and persisting until menopause, suggest the involvement of sex hormone variations and the gonadal hormones' modulatory effects on brain circuits.^26^, ^27^, ^28^ Oestrogen, for example, is known to promote cognitive functions, modulate gene transcription, neurotrophin development, and influence catecholamine and monoamine metabolism and neurotransmission.^29^ In healthy adult women, the menstrual cycle stage significantly affects the activation level of the brain's reward and stress response networks.^30^, ^31^ Lower blood estradiol levels and higher serum progesterone levels, associated with hypoactivation of the brain's physiological stress circuits, have been observed in women with MDD compared to healthy controls.^32^ Furthermore, monoamine oxidase (A), an enzyme involved in oxidative stress, apoptosis and monoamine metabolism, increases in the brain during perimenopause. This increase is linked to estradiol variations during perimenopause and contributes to susceptibility.^33^ Conversely, testosterone may contribute to increased resilience in males.^20^ Testosterone is strongly positively correlated with the extension of social connections, success‐related emotions and social dominance.^34^ Notably, testosterone levels decrease following stress,^35^ and individuals with PTSD or MDD often exhibit low circulating levels of this hormone.^36^, ^37^ Preliminary research suggests that testosterone supplementation increasing lean body mass, improving body strength and improve exercise‐induced coronary ischemia.^38^ in conjunction with selective serotonin reuptake inhibitors like fluoxetine, paroxetin and sertaline^39^ may benefit men with treatment‐resistant depression.^37^

These insights collectively underscore the complex role of neuroendocrine systems in modulating stress resilience and susceptibility to mood disorders. The intricate interactions between these systems offer promising avenues for developing targeted therapeutic strategies and enhancing our understanding of the biological underpinnings of stress‐related psychopathology.

## 4 ADVANCEMENTS IN UNDERSTANDING HPA AXIS DYNAMICS IN STRESS RESILIENCE: INSIGHTS FROM ANIMAL MODELS

**FIGURE 5:**
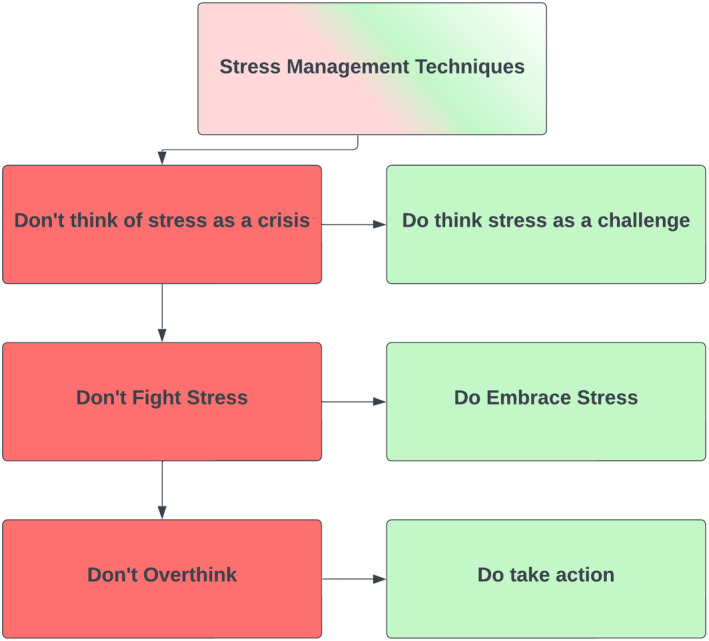
Role in stress response.

Recent studies exploring the molecular underpinnings of resilience in relation to the hypothalamo–pituitary–adrenal (HPA) axis have predominantly utilized animal models, particularly focusing on developmental stress scenarios. A notable observation is that adult rats exposed to stress inoculation via postnatal handling demonstrate a distinct neuroendocrine profile compared to their unstressed counterparts or those subjected to maternal separation. These differences include lower basal levels of corticotropin‐releasing factor (CRF), attenuated responses of CRF, adrenocorticotropic hormone (ACTH) and corticosterone to stress, and a more rapid reversion to basal stress hormone levels following stress exposure.^40^ Maternal care behaviour has emerged as a critical determinant of resilience to early‐life stress, leading to significant individual differences in gene expression and subsequent activation of the neuroendocrine stress response.^41^ Studies show that maternal behaviours such as grooming, licking and arched‐back nursing in rats are more pronounced in mothers of handled offspring compared to non‐handled ones.^42^ Intriguingly, there is an inverse correlation between the extent of these maternal behaviours and the levels of stress‐induced plasma ACTH and corticosterone in the adult offspring.^42^ The neuroendocrine basis of this resilience has been traced to enhanced expression of hippocampal glucocorticoid receptors (GRs) in offspring receiving higher levels of maternal care. This upregulation heightens the sensitivity to the glucocorticoid‐mediated negative feedback on CRF and arginine vasopressin (AVP) secretion. Further research has identified various molecular factors contributing to this increased GR expression in handled pups. These include elevated secretion of thyroid hormones, increased serotonin levels across the hippocampus, and heightened expression of the cAMP‐inducible transcription nerve growth factor inducible protein A (NGFI‐A), which binds to exon 17 of the GR promoter^41^, ^42^, ^43^, ^44^ (Figure 5).

Epigenetic mechanisms in adult rats have been shown to maintain glucocorticoid receptor sensitivity in stress‐resilient animals. For instance, the NGFI‐A consensus sequence on the GR gene promoter is predominantly methylated in offspring of low licking and grooming (LG) mothers, while it is associated with acetylated histone H3 in offspring of high LG mothers.^41^ Methylation at this site inhibits NGFI‐A binding to the GR promoter, contrasting with the effects of acetylation. Thus, high LG maternal care leads to enduring epigenetic modifications that enhance glucocorticoid negative feedback sensitivity, increased glucocorticoid receptor expression, reduced CRF and AVP release from the hypothalamus, and ultimately, a diminished HPA axis response to future stressors.^45^

Additionally, recent studies have identified critical mediators in the modulation of the HPA axis response to adult stress, despite gaps in our understanding. These include CRF regulation of brain‐derived neurotrophic factor (BDNF) expression and pro‐resilience epigenetic changes at the CRF gene in paraventricular nucleus (PVN) neurons. In mice exhibiting susceptibility to chronic social defeat stress (CSDS), researchers noted elevated CRF mRNA expression in the PVN and reduced methylation at specific CpG sites of the CRF promoter.^46^ Viral suppression of CRF in the PVN following social defeat revealed resilient behaviour, as indicated by the social interaction test, suggesting that CRF promoter methylation leads to adaptive neuroendocrine and behavioural responses in resilient animals.^46^

**FIGURE 6:**
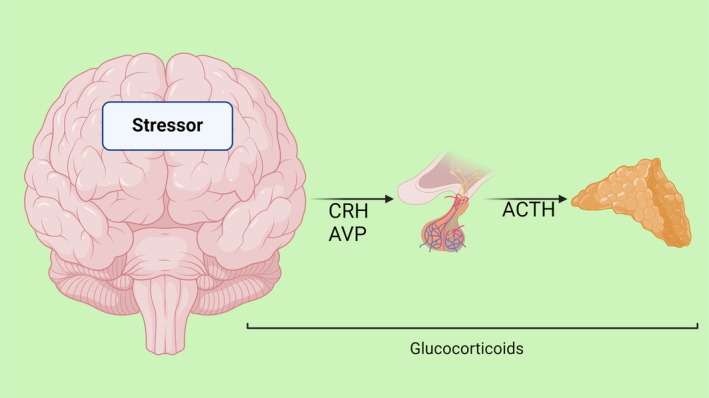
Comparison of basal (Top) and stress‐induced (Bottom) HPA‐axis function: hypothalamic GR mRNA, CRHR2 mRNA expression and amygdala GR mRNA.^49^, ^50^

Further insights were provided by a study demonstrating that optogenetic stimulation of phasic firing in the dopaminergic neurons of the ventral tegmental area (VTA) facilitated social avoidance behaviour in mice following subthreshold social defeat stress.^47^ This effect was mediated by CRF‐gated BDNF expression in the NAc, a target region for VTA dopaminergic projections. The inhibition of social avoidance effects by CRF antagonist infusion alleviate development of anxiety like behaviour and stress related alterations of gut functions, increase in lean body mass, improvement in body strength, Ca improved exercise induced coronary ischemia^48^ underscores CRF as a key modulator of susceptibility and resilience in coping with stress. Future research aims to elucidate variations in CRF activity in the NAc among resilient subjects, further advancing our understanding of the neuroendocrine dynamics in stress resilience (Figure 6).

## 5 ELUCIDATING THE MESOCORTICOLIMBIC REWARD CIRCUITRY IN CNS RESILIENCE: MOLECULAR AND CELLULAR DYNAMICS

A substantial body of contemporary research in neurobiology focuses on unravelling the central mechanisms of resilience within the brain, particularly emphasizing the mesocorticolimbic reward circuitry. This circuitry is fundamental to the brain's adaptive function of directing attention towards the acquisition of natural rewards, a process essential for survival.^49^ The mesocorticolimbic system comprises an intricate network of neurons originating from various brain regions, including the nucleus accumbens (NAc), medial prefrontal cortex (mPFC), ventral tegmental area (VTA), amygdala, hippocampus, lateral habenula and hypothalamus. Collectively, these regions orchestrate a range of cognitive and psychological functions like decision making, fleeing^51^ that are often disrupted by stress in individuals with depression or anxiety.^52^ The mesocorticolimbic connections are diverse and complex. This discussion primarily focuses on the well‐characterized pathways of the VTA‐NAc reward circuit. The ventral striatum's medium spiny neurons (MSNs), which are GABAergic, receive inputs from dopaminergic neurons in the VTA. Dopamine release from VTA neurons is triggered in response to reward‐related stimuli, initiating behaviours such as eating, and sometimes in reaction to aversive stimuli. The NAc utilizes two distinct pathways involving D1‐type MSNs for the direct pathway and D2‐type MSNs for the indirect pathway. These routes enable the NAc to establish bidirectional connections with the VTA. The indirect pathway involves GABAergic interneurons within the ventral pallidum, which then form connections with VTA neurons.^52^

**FIGURE 7:**
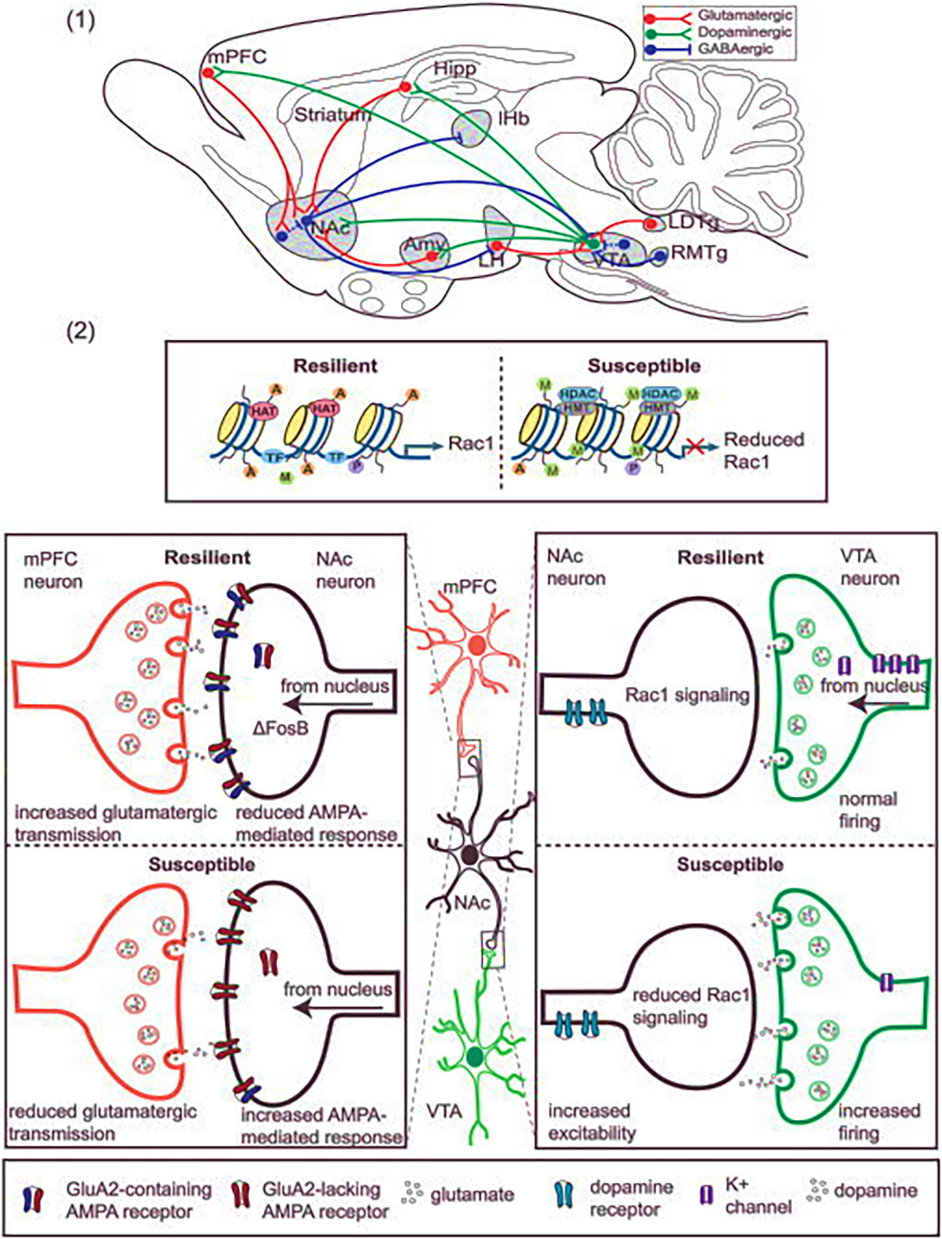
Elucidating the mesocorticolimbic reward circuitry in CNS resilience: molecular and cellular dynamics and a brief history of resilience research.^49^, ^57^, ^58^

Human neuroimaging studies provide support for the involvement of the VTA‐NAc pathway in the pathophysiology of depression, although results have been somewhat mixed. These studies have demonstrated consistent reductions in NAc volume in older patient populations and decreases in NAc activity among adult patients suffering from depression.^53^, ^54^, ^55^, ^56^ These observations underline the critical role of the mesocorticolimbic reward circuitry in modulating emotional and cognitive responses to stress, thereby contributing to resilience or vulnerability in the context of mood disorders (Figure 7; Table 1).

**TABLE 1:** Stress‐induced detrimental effects on CNS.

Functional aspects	Site involved	Conformational changes	Responsive alterations
Memory	Hippocampus (Glucocorticoid receptors)	Neurogenesis as well as atrophy disorders,^1^ decreased dendritic branching,^59^ reducing the number of neurons as well as modifying synaptic terminals,^60^ reducing neurogenesis in hippocampus,^61^ reduction of hippocampus volume,^62^ modifying LTP^63^	Problems regarding memory consolidation,^1^ decrease in spatial memory,^64^ decline in verbal memory, disruption in hippocampus dependent loading data^62^
	Amygdala (Noradrenaline)		
Cognition and learning	Hippocampus, amygdala and temporal lobe	Neurodegenerative processes activation^65^	Reducing of cognition,^66^ creating psychiatric, cognitive and behavioural disorders,^65^ hippocampus‐related cognitive impairments,^67^ reducing the response time^68^

## 6 EMERGING STIMULATION TECHNIQUES TARGETING THE VENTRAL TEGMENTAL AREA AND NUCLEUS ACCUMBENS CIRCUITRY

**FIGURE 8:**
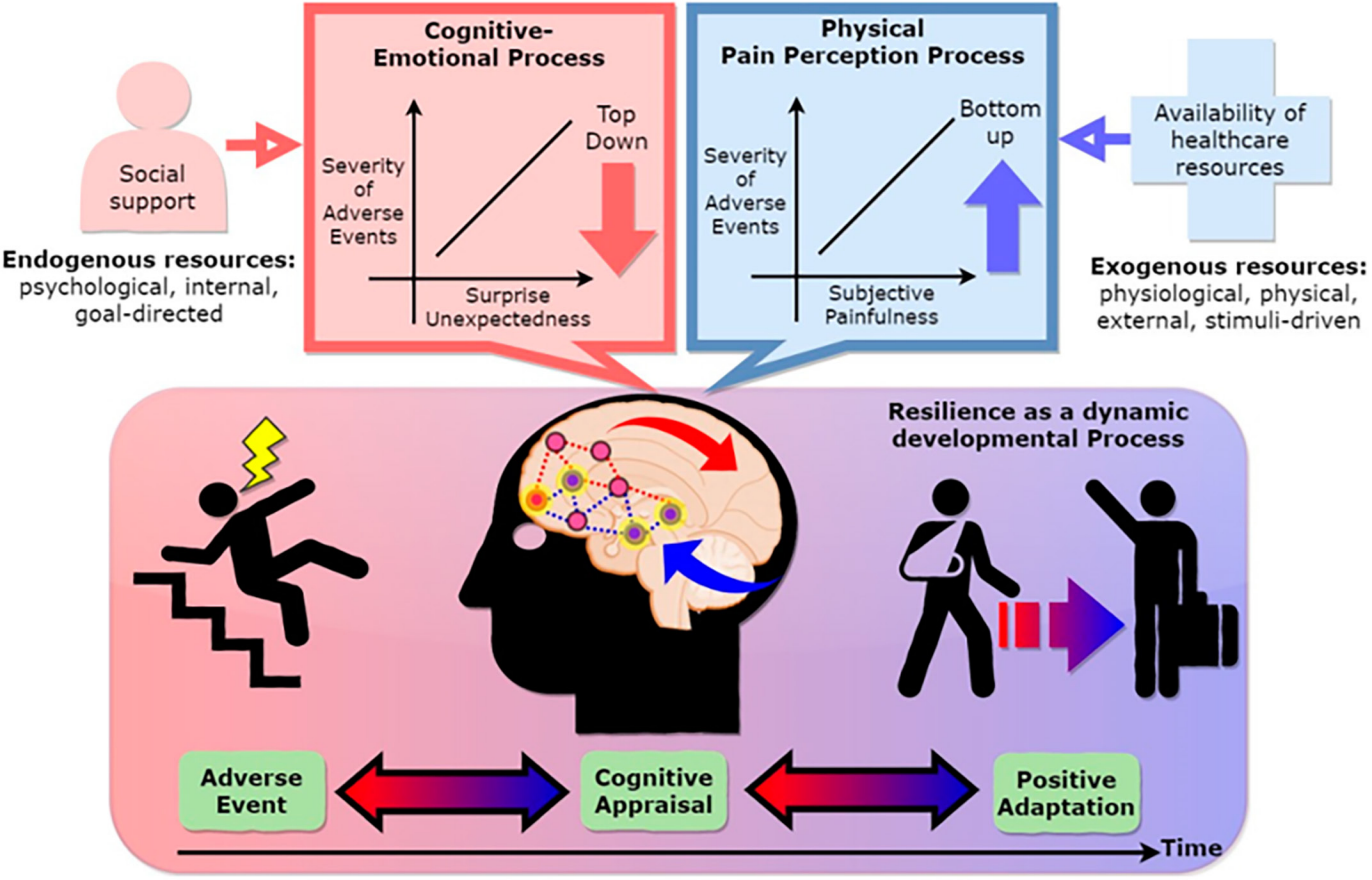
Mechanistic pathway of resilience.^70^

Recent advancements in neuromodulation have introduced novel stimulation techniques that specifically target the VTA connection like optogenic simulation^69^ to the nucleus accumbens (NAc) and its efferent pathways (as communicated by T. Schlaepfer). These techniques are at the forefront of exploring the therapeutic potential of directly modulating key nodes within the mesocorticolimbic circuit, which plays a crucial role in reward processing and mood regulation. The primary focus of these interventions is on the VTA‐NAc axis, a central component of the brain's reward system. By stimulating this pathway, researchers aim to influence the neural dynamics associated with various psychiatric conditions, particularly depression and anxiety disorders, where dysregulation in this circuitry is often implicated. However, the specific effects of these stimulant techniques on the microcircuitry of the NAc remain an area of ongoing research. One critical question pertains to the extent to which these interventions influence not only the targeted area but also engage other neural pathways and synapses. Particularly, there is a keen interest in understanding whether the stimulation of the NAc inadvertently activates adjacent fibres and pathways that project beyond the immediate region of the NAc. Such collateral activation could potentially broaden the impact of these techniques, offering insights into the interconnected nature of neural networks involved in mood and reward processing. The exploration of these stimulation methods holds promise for refining our understanding of the brain's reward system and developing more precise therapeutic interventions for neuropsychiatric disorders. Continued research in this area is essential to elucidate the broader implications of targeted neural stimulation and its effects on the complex web of synaptic connections within the brain (Figure 8).

## 7 TRADITIONAL HERBAL MEDICINE IN MENTAL HEALTH MANAGEMENT: A DETAILED PERSPECTIVE

The role of traditional herbal medicine in enhancing physical and mental well‐being has gained increasing recognition, especially in the context of managing mental health disorders.^71^, ^72^, ^73^ The growing preference for herbal remedies over conventional pharmacological treatments is often attributed to concerns regarding the adverse effects and negative responses associated with synthetic chemical medications like dry mouth, cramps, sleep problems and tardive dyskynesia.^74^, ^75^, ^76^ This shift reflects a broader trend towards holistic and integrative approaches in healthcare. Anxiety, a prevalent mental health condition, manifests through a spectrum of symptoms that can significantly impair daily functioning. These symptoms include muscle tension, trembling, accelerated heartbeat and breathing, headaches, dry mouth, difficulty swallowing, excessive sweating, abdominal pain, dizziness, blurred vision, diarrhoea, frequent urination, irritability, quick temper, sexual dysfunction, diminished concentration, sleep disturbances and nightmares. In response to these varied manifestations, traditional herbal medicine offers a range of natural remedies that have been evaluated for their therapeutic potential. Extensive research has identified several herbs with promising psycho‐oncologic benefits in mitigating symptoms of anxiety and depression. Notable among these are saffron, chasteberry, lavender, chamomile, passionflower and sage.^77^ These herbs have been found to exert calming effects, reduce stress and improve mood, making them viable complementary options in mental health treatment.

Additionally, certain nutrients play a critical role in stress regulation and overall mental health. These include amino acids such as tryptophan, phenylalanine, tyrosine, and theanine, as well as essential vitamins and minerals like vitamins B and C, selenium and magnesium. The inclusion of complex carbohydrates like breakfast cereals, whole grain bread^78^ in the diet also contributes to stress management.^79^, ^80^ These nutrients are integral to various biochemical pathways in the brain that influence mood and cognitive functions. Traditional herbal medicine, complemented by a diet rich in specific nutrients, presents a holistic approach to managing mental health (Table 2). This approach underscores the importance of natural substances in modulating psychological well‐being and offers a gentler alternative to conventional pharmacological interventions. As research in this field expands, it is anticipated that more comprehensive and personalized herbal and nutritional strategies will emerge, further enhancing the efficacy of mental health treatments.

**TABLE 2:** Herbal plants and its benefits.^81^, ^82^, ^83^, ^84^, ^85^, ^86^, ^87^, ^88^, ^89^, ^90^, ^91^, ^92^, ^93^, ^94^, ^95^, ^96^, ^97^, ^98^, ^99^, ^100^, ^101^, ^102^, ^103^, ^104^, ^105^, ^106^, ^107^, ^108^, ^109^, ^110^, ^111^, ^112^, ^113^, ^114^, ^115^, ^116^, ^117^, ^118^, ^119^, ^120^, ^121^

Herbal plant	Family	Key points
Ginseng (Panax ginseng)	Araliaceae	By blocking hypothalamo–pituitary–adrenal axis mechanism, Panax ginseng extract provides antidepressant‐like effects In people who are under a lot of stress, Korean red ginseng may assist in balancing sympathetic nervous system as well as enhance cognition. Ginseng outperforms other adaptogens in its ability to control stress For the treatment of menopausal depression, white ginseng may be a trustworthy all‐natural substitute for antidepressant medications
Herbal tea		It could raise one's quality of life and lessen the intensity of insomnia symptoms
Scullcap (*Scutellarialateriflora*)	Lamiaceae	It is frequently used to treat anxiety
Chamomile (Matricariarecutita)	Asteraceae	It has no negative side effects and helps lessen anxiety symptoms
*Echinaceae purpurea*	Asteraceae	It may lessen anxiety
Passionflower (Passiflora incamata)	Passifloraceae	It can considerably lessen the signs of anxiety
Saffron (Crocus sativus)	Iridaceae	Its stamen and petals might lessen the symptoms of sadness. It possesses anxiolytic, serotonin reuptake inhibition, dopamine, norepinephrine and other actions
St John, s wort: SJW (Hypericum perforatum)	Hypericaceae	It has antidepressant and sedative properties
Butea superba	Leguminosae	Improve emotional and cognitive deficiencies
Bacopa monnieri	Plantaginaceae	Remediate emotional and cognitive deficiencies
Dracocephalummoldavica L. (Lamiaceae)	Lamiaceae	It is an Asian native, and an aqueous extract of it has been shown to provide anti‐anxiety‐like properties.
Foeniculum vulgare Mill (Apiaceae)	Apiaceae	The elevated plus maze as well as open field experiments using the essential oils isolated by aerial portions revealed anxiolytic‐like effects, and it is hypothesized that this may help to mediate the anxiolytic‐like activity.
Lavender (Lavandula spp.)	Lamiaceae	It lowers anxiety and depressive symptoms and modulates GABA
Persian Lavender (Nepeta menthoides)	Lamiaceae	Persian lavender, also known as Ustukhuddus, is a herb from the Lamiaceae family that has traditionally been used to treat severe depressive disorder in patients The extract's polyphenol and flavonoid concentration may have contributed to its therapeutic benefits
Rhodiola rosea extract (RRE)	Crassulaceae	Rosen root provides thorough therapy for stress symptoms that can ward off long‐term stress, despair, and issues linked to stress Nitric oxide, stress‐induced protein kinases and cortisol release inhibition are roseroot's three key properties.

## 8 MITOCHONDRIAL OXIDATIVE PHOSPHORYLATION AND ITS ROLE IN FREE RADICAL FORMATION AND OXIDATIVE STRESS

Mitochondrial oxidative phosphorylation (OXPHOS) is a critical biological process intricately linked to the formation of reactive oxygen species (ROS), which play a pivotal role in both physiological functions and pathophysiological conditions. Oxidative stress, defined as the imbalance between ROS production and antioxidant defences, results when the generation and accumulation of ROS exceed the body's capacity to neutralize and eliminate them.^122^ While ROS, including superoxide anion (O2•) and hydroxyl radical (OH•), are essential for normal cellular signalling,^123^ their overproduction can lead to cellular and tissue damage by directly harming biological molecules such as lipids, proteins and nucleic acids.^122^, ^124^

**FIGURE 9:**
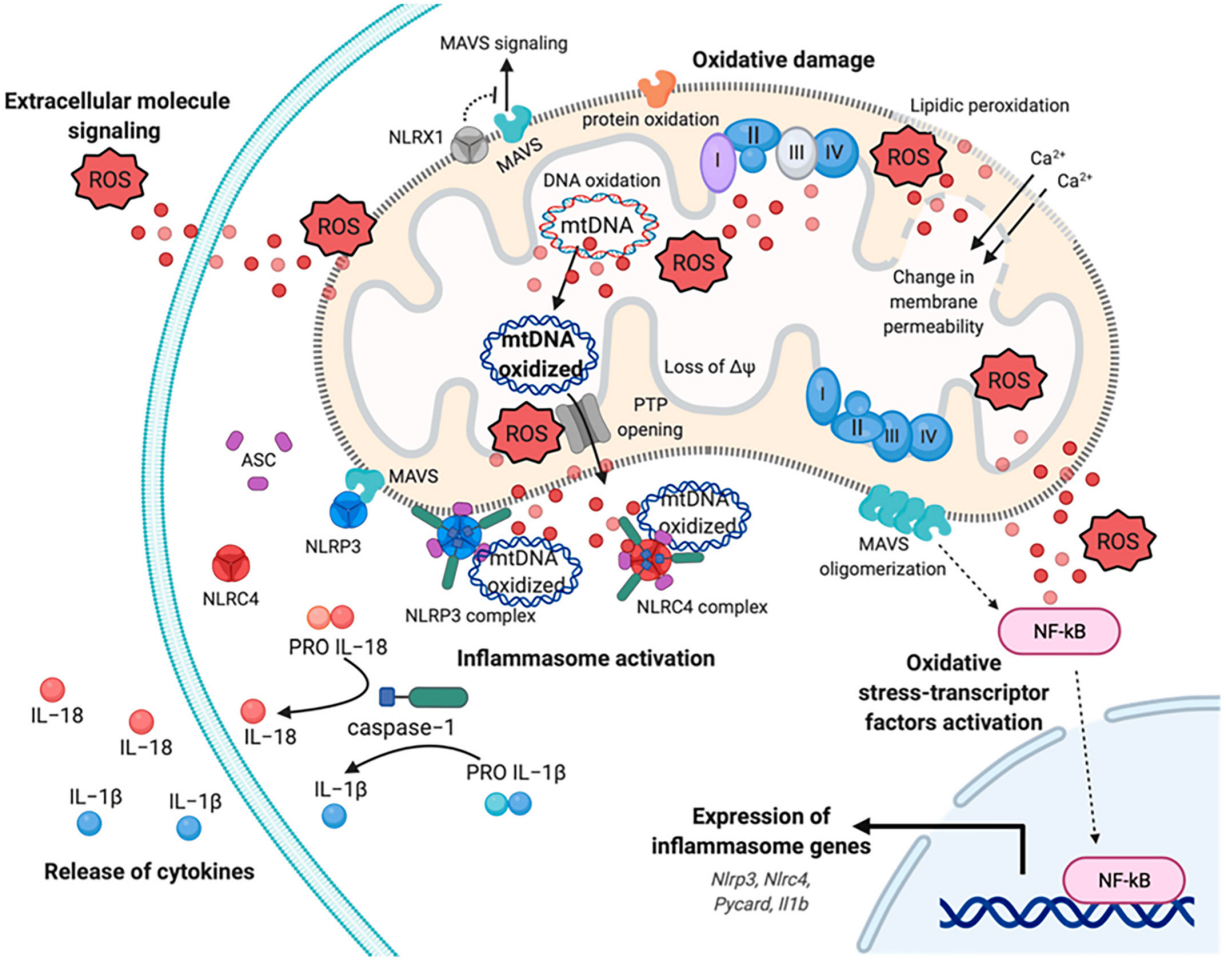
Mechanism of mitochondria‐mediated signalling in innate immunity.^136^

Exogenous factors contributing to oxidative stress include ionizing radiation (such as X‐rays and UV rays), environmental pollution, smoking, exposure to heavy metals and certain medications. Endogenously, ROS are primarily generated as byproducts of cellular oxygen metabolism.^125^ Chronic inflammation further exacerbates ROS production, amplifying oxidative stress within the body.^126^ The superoxide anion and the hydroxyl radical are among the most reactive free radicals. The hydroxyl radical, characterized by its unstable nature and unpaired electrons, can initiate oxidation processes leading to the formation of secondary ROS‐like hydrogen peroxide (H_2_O_2_), peroxynitrite (ONOO‐) and hypochlorous acid (HOCl).^125^ Mitochondria, the cellular powerhouses, are the primary source of free radicals in cells. Beyond their fundamental role in ATP production, mitochondria are involved in lipid synthesis, heme and purine production, steroidogenesis, and regulate intracellular calcium (Ca^2+^) homeostasis, thermogenesis, cell growth and apoptosis.^50^, ^127^, ^128^ During the intense oxidative metabolism in mitochondria, ROS are generated, constituting approximately 1%–2% of the total molecular oxygen consumed during normal respiration. Notably, the majority of free radicals, especially the superoxide anion, are byproducts of mitochondrial respiration occurring during electron transfer across complexes I, II and III of the mitochondrial electron transport chain.^129^, ^130^ Mitochondria are estimated to contain 5‐ to 10‐times more oxygen than the cytosol or nucleus, making them significant sites for ROS generation.^131^ Factors such as hypoxia, nutrient availability, inflammatory cytokines and changes in mitochondrial membrane potential can provoke oxidative damage in mitochondria.^127^, ^132^ Excessive ROS formation or impaired mitochondrial antioxidant defence can lead to damage to mitochondrial DNA, proteins and lipids, potentially impairing mitochondrial function and triggering cell death via the release of pro‐apoptotic factors from the mitochondrial intermembrane space. Mitochondria produce adenosine triphosphate (ATP) via oxidative phosphorylation. Structurally, they consist of two membranes: an inner membrane, which forms the mitochondrial cristae, and an outer membrane that separates the mitochondria from the cytoplasm. The outer membrane encloses the mitochondrial matrix, housing mitochondrial DNA (mtDNA).^133^ Oxidative phosphorylation occurs on the inner membrane, where four large multi‐subunit enzyme complexes are located: NADH: ubiquinone oxidoreductase (complex I), succinate dehydrogenase (complex II), coenzyme Q: cytochrome c reductase (complex III) and ATP synthase (complex V).^134^ These complexes constitute the electron transport chain, which facilitates electron transfer through a series of redox reactions, beginning with reduced nucleotides NADH and FADH2 and culminating with oxygen as the final electron acceptor, resulting in water (H_2_O) formation. The energy released in this process creates a proton gradient used by ATP synthase to phosphorylate ADP, producing ATP^134^, ^135^ (Figure 9).

## 9 PROTACs: EMERGING THERAPEUTICS AND THEIR POTENTIAL INTERPLAY WITH MITOCHONDRIAL OXIDATIVE PHOSPHORYLATION IN MANAGING OXIDATIVE STRESS

**FIGURE 10:**
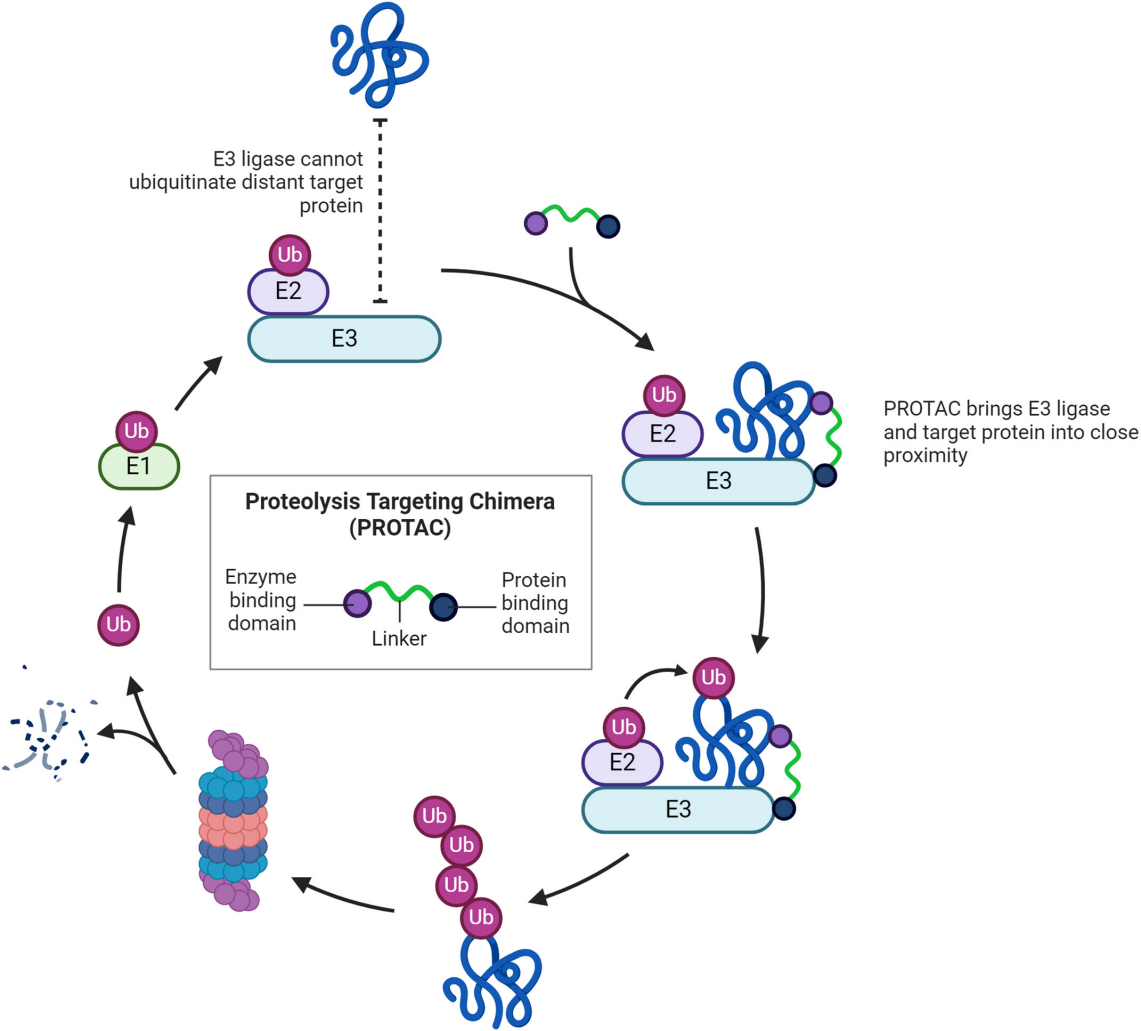
Mitochondrial oxidative phosphorylation to PROTACs in addressing oxidative stress‐related pathologies (Created with Biorender.).

Proteolysis targeting chimeras (PROTACs) represent a cutting‐edge advancement in the field of targeted protein degradation, offering a novel approach to therapeutic intervention.^137^, ^138^, ^139^ PROTACs are bifunctional molecules designed to induce selective degradation of specific proteins. They function by creating a ternary complex with the target protein and an E3 ubiquitin ligase, leading to the ubiquitination and subsequent proteasomal degradation of the target protein. This targeted degradation presents a significant leap from traditional inhibitor‐based therapies, as PROTACs can eliminate both the enzymatic and nonenzymatic functions of their targets (Figure 10). The relevance of PROTACs in the context of OXPHOS is particularly intriguing. Oxidative stress, primarily driven by an imbalance in ROS production and antioxidant defences, is a contributing factor to various pathologies, including neurodegenerative diseases, cancer and cardiovascular disorders. The excessive generation of ROS, as a byproduct of mitochondrial respiration, especially in the electron transport chain's complexes I, II and III, can lead to significant cellular damage.^129^, ^130^ PROTACs could potentially be engineered to selectively degrade proteins that are either directly involved in excessive ROS production or impaired in conditions of oxidative stress. For instance, targeting proteins that abnormally elevate mitochondrial respiration or disrupt electron transport chain efficiency could mitigate the overproduction of ROS, thereby alleviating oxidative damage. Moreover, PROTACs could be utilized to modulate the cellular antioxidant response. Proteins involved in the regulation of antioxidant defence mechanisms, if dysregulated, can contribute to an imbalance in ROS levels. By selectively degrading such regulatory proteins, PROTACs could restore the equilibrium between ROS production and antioxidant defences, potentially offering a novel approach to managing conditions associated with oxidative stress. The application of PROTACs in targeting key mitochondrial proteins could also extend to regulating apoptosis, a process intimately linked with mitochondrial function. In conditions of excessive ROS production and consequent mitochondrial damage, apoptosis can be aberrantly activated, leading to pathological cell death. PROTACs could be designed to target and degrade proteins that trigger this process inappropriately, thus offering a strategy to prevent cell loss in diseases where apoptosis is dysregulated.

PROTACs present a promising therapeutic strategy with potential applications in managing oxidative stress and its associated pathologies. Their ability to precisely target and degrade specific proteins could be harnessed to modulate mitochondrial function, particularly in the context of oxidative phosphorylation and ROS management. As research in this field progresses, PROTACs could emerge as a key tool in addressing the challenges^140^, ^141^ posed by diseases linked to mitochondrial dysfunction and oxidative stress.

## 10 DISCUSSION: INTEGRATING INSIGHTS FROM MITOCHONDRIAL OXIDATIVE PHOSPHORYLATION TO PROTACs IN ADDRESSING OXIDATIVE STRESS‐RELATED PATHOLOGIES

This paper provides a comprehensive exploration of the intricate mechanisms of mitochondrial oxidative phosphorylation (OXPHOS) and its role in the generation of reactive oxygen species (ROS), which are central to the concept of oxidative stress. Oxidative stress, arising from an imbalance between ROS production and antioxidant defences, has been implicated in a myriad of pathologies, including neurodegenerative diseases, cancer and cardiovascular conditions. The discussion has highlighted the dual nature of ROS—as necessary signalling molecules at physiological levels and as agents of cellular damage when in excess. This dichotomy underscores the importance of maintaining a delicate balance between ROS generation, primarily during mitochondrial respiration, and the body's antioxidant mechanisms. The focus then shifted to proteolysis targeting chimeras (PROTACs), a revolutionary approach in targeted protein degradation, which opens up new vistas in therapeutic interventions. PROTACs, by facilitating the selective degradation of specific proteins, offer a targeted strategy to potentially modulate proteins involved in ROS production or the antioxidant response. Their application could extend to influencing mitochondrial function directly, especially considering the central role of mitochondria in both energy production and as the primary source of cellular ROS. The potential of PROTACs to target proteins involved in the electron transport chain or those regulating mitochondrial dynamics and apoptosis presents a novel approach to managing oxidative stress at its cellular source. The interplay between mitochondrial dysfunction and oxidative stress forms a critical nexus in the pathology of various diseases. By understanding the molecular underpinnings of mitochondrial OXPHOS and the generation of ROS, there is an opportunity to strategically target these pathways using PROTACs. This could involve downregulating proteins that contribute to excessive ROS production or upregulating proteins that enhance cellular antioxidant capacity. Moreover, the ability of PROTACs to target and degrade specific proteins offers a more nuanced approach than traditional therapeutics, which often have broader effects and associated side effects.

In conclusion, the integration of insights from mitochondrial oxidative phosphorylation with the emerging field of PROTACs provides a promising avenue for developing novel therapeutic strategies against diseases associated with oxidative stress. This approach not only offers a more precise understanding of the molecular mechanisms involved in disease pathology but also opens up the possibility of more targeted and effective treatments. Future research in this area is poised to uncover further connections between mitochondrial function, oxidative stress and the potential of PROTACs in disease management, marking an exciting frontier in biomedical research.

## 11 FUTURE DIRECTIONS

This exhaustive journal piece underscores the intricacy and systemic character of stress resistance, suggesting potential directions for further investigation and therapeutic strategies.^20^ The study emphasizes the necessity for a multifaceted strategy that combines perspectives from neuroendocrinology, cellular biology, psychology and even incorporates elements of traditional medicine.^41^ A notable inference involves the possibility of utilizing focused treatments such as PROTACs to selectively adjust proteins and pathways associated with dysfunctional reactions to stress.^137^ Subsequent investigations might delve into PROTACs designed to target the HPA axis, inflammatory processes, mitochondrial irregularities or pathways related to oxidative stress. Precision targeting of particular proteins associated with the dysregulation of the stress system holds potential for enhancing the efficacy of therapeutic interventions.^128^

Furthermore, the knowledge gained from animal models regarding the distinctions in epigenetic, neuroendocrine and behavioural aspects between stress resilience and susceptibility lays the groundwork for translational research in humans.^46^ Subsequent investigations could examine these biomarkers in individuals experiencing anxiety, depression and PTSD to reveal novel targets for therapeutic interventions.^121^ The study further underscores the importance of addressing psychological and lifestyle elements, not solely focusing on biological mechanisms. A holistic approach that combines pharmacotherapies with behavioural interventions, stress inoculation, mindfulness practices, exercise, nutrition and traditional herbal medicine may yield more significant benefits.^12^ Comprehensive treatment approaches involving multiple strategies should undergo evaluation in clinical trials.^73^ Ultimately, the results indicate that we are in the initial phases of unravelling the intricacies of stress resilience. Advancing research collaborations spanning neuroscience, psychology, cell biology, genetics and other disciplines are imperative to cultivate a comprehensive, systems‐level understanding of resilience.^7^ Such collaborative efforts have the potential to pave the way for personalized and efficacious approaches to stress management in the future.

## 12 CONCLUSION

The COVID‐19 pandemic has significantly impacted mental health globally, affecting members of the public, patients, medical professionals, children and the elderly. The extended duration of the pandemic and associated self‐isolation measures have led to a decline in sleep quality and an increase in stress levels, contributing to heightened risks of depression, anxiety and even suicidal ideation. These psychological effects, compounded by the stress of living through a global health crisis, can exacerbate secondary diseases and disorders of the immune system like multiple mylemoma, viral hepatitis.^142^ In this context, the importance of maintaining mental and physical health becomes paramount. Key strategies for patients and the general population include a healthy diet, regular breaks from work, adequate sleep and relaxation techniques. In addition to these, the incorporation of traditional herbal remedies into daily routines offers a complementary approach to enhancing overall health and well‐being. Herbs such as lavender, chamomile, ginseng, saffron, passionflower, cardamom, kava and Chinese dates, along with traditional formulas like Dan‐zhi‐xiao‐yaosan, Sinsian, So‐ochim‐tang‐gamiband and Yokukansan, have shown promise in treating anxiety, mental health issues and stress. These natural remedies can be an integral part of a holistic health care approach, particularly in these challenging times.

The role of mitochondrial OXPHOS in the generation of ROS and the innovative potential of PROTACs offer a transformative approach in understanding and treating diseases related to oxidative stress. The selective degradation of specific proteins by PROTACs presents a targeted strategy to potentially modulate proteins involved in ROS production or the antioxidant response, thereby providing a novel approach to managing conditions associated with oxidative stress. Looking to the future, research should continue to explore the interplay between mitochondrial function, oxidative stress, PROTACs and traditional herbal remedies in the context of mental health, particularly considering the ongoing challenges posed by the COVID‐19 pandemic. Further studies are needed to understand the long‐term psychological impacts of the pandemic and to develop effective strategies for mitigating these effects. Additionally, the continued investigation into the molecular mechanisms of diseases associated with oxidative stress and the development of targeted therapies using PROTACs will be crucial in advancing our understanding and treatment of these conditions. The integration of mitochondrial insights, the potential of PROTACs and the use of traditional herbal medicine presents a multifaceted approach to addressing both the physical and psychological challenges of our times. As research progresses, it is anticipated that these combined strategies will lead to more effective treatments and improved patient outcomes in a wide range of diseases, particularly those exacerbated by the stress and challenges of the COVID‐19 pandemic.

## AUTHOR CONTRIBUTIONS

**Arghya Bhattacharya:** Conceptualization (equal); formal analysis (equal); methodology (equal); writing – original draft (equal). **Manas Chakraborty:** Investigation (equal); supervision (equal); writing – review and editing (equal). **Ananya Chanda:** Formal analysis (equal); methodology (equal); resources (equal); visualization (equal); writing – original draft (equal). **Taha Alqahtani:** Formal analysis (equal); supervision (equal); writing – review and editing (equal). **Ajoy Kumer:** Conceptualization (equal); methodology (equal); project administration (equal); writing – original draft (equal). **Bikram Dhara:** Project administration (equal); supervision (equal); writing – review and editing (equal). **Moitreyee Chattopadhyay:** Conceptualization (equal); investigation (equal); resources (equal); supervision (equal); visualization (equal); writing – review and editing (equal).

## CONFLICT OF INTEREST STATEMENT

The authors declare have no conflict of interest and the manuscript which are submitted not present in anywhere.

## Data Availability

No data are available.
